# Alternative Sources of *n*-3 Long-Chain Polyunsaturated Fatty Acids in Marine Microalgae

**DOI:** 10.3390/md11072259

**Published:** 2013-06-27

**Authors:** Dulce Alves Martins, Luísa Custódio, Luísa Barreira, Hugo Pereira, Radhouan Ben-Hamadou, João Varela, Khalid M. Abu-Salah

**Affiliations:** 1 Centre of Marine Sciences, University of Algarve, Faro 8005-139, Portugal; E-Mails: alvesmartinsd@gmail.com (D.A.M.); lcustodio@ualg.pt (L.C.); lbarreir@ualg.pt (L.B.); hgpereira@ualg.pt (H.P.); bhamadou@ualg.pt (R.B.-H.); 2 King Abdullah Institute for Nanotechnology, King Saud University, Riyadh 11451, Saudi Arabia

**Keywords:** marine microalgae, *n*-3 long-chain polyunsaturated fatty acids, EPA, DHA

## Abstract

The main source of *n*-3 long-chain polyunsaturated fatty acids (LC-PUFA) in human nutrition is currently seafood, especially oily fish. Nonetheless, due to cultural or individual preferences, convenience, geographic location, or awareness of risks associated to fatty fish consumption, the intake of fatty fish is far from supplying the recommended dietary levels. The end result observed in most western countries is not only a low supply of *n*-3 LC-PUFA, but also an unbalance towards the intake of *n*-6 fatty acids, resulting mostly from the consumption of vegetable oils. Awareness of the benefits of LC-PUFA in human health has led to the use of fish oils as food supplements. However, there is a need to explore alternatives sources of LC-PUFA, especially those of microbial origin. Microalgae species with potential to accumulate lipids in high amounts and to present elevated levels of *n*-3 LC-PUFA are known in marine phytoplankton. This review focuses on sources of *n*-3 LC-PUFA, namely eicosapentaenoic and docosahexaenoic acids, in marine microalgae, as alternatives to fish oils. Based on current literature, examples of marketed products and potentially new species for commercial exploitation are presented.

## 1. Introduction

Research on microalgal lipids has gathered significant interest over the last few years, not only due to their bioactive properties, but also as an important component of aquaculture feed, feedstock for the production of biofuels, and energy and biomass transfer between different trophic levels in the global food web [1,2,3]. This review will focus on the long-chain polyunsaturated fatty acids (LC-PUFA), their role in promoting human health or disease, and the dire need for alternative sources of LC-PUFA able to replace fish meal/oil as the bulk provider for this important class of biochemicals. Although a few topics have been covered elsewhere [4,5,6,7], this review integrates the current knowledge on microalgal LC-PUFA biosynthesis and the use of specific examples of commercial photo- and heterotrophic microalgae for the production of fatty acids (FA) that can have an impact on the health of humans and other vertebrates. Lastly, this review discusses the methods usually employed in LC-PUFA quantitation and the care needed for preventing LC-PUFA depletion upon microalgal cell disruption, which could lead to incorrect fatty acid profiles.

## 2. Long Chain Polyunsaturated Fatty Acids and Their Importance in Human Health

The importance of diet to physical and mental wellbeing has gained increased attention from the medical community and the public in the last decades. The dietary intake of LC-PUFA, particularly the *n*-3 series eicosapentaenoic (EPA) and docosahexaenoic acids (DHA), occurs mainly through seafood consumption, and is typically low in the so-called “western” diet [8]. The relevance of these nutrients was first highlighted when populations with frequent seafood consumption habits (e.g., Inuits) were noted to present a lower incidence of cardiovascular diseases (CVD) relative to others consuming less seafood and more *n*-6 PUFA, C_18_ fatty acids, mainly linoleic acid (LA) [9,10]. At present, most western populations consume an average of less than 0.5 g of *n*-3 LC-PUFA daily, lower than values determined for Japan and Norway (1–3 g daily), and much lower than the intake of these nutrients by Inuit populations, which amount to 10–14 g day^−1^ [8]. Most health benefits of seafood consumption are attributed to EPA and DHA. Nonetheless, these foods are also rich sources of other nutrients, such as high quality protein, various vitamins (e.g., A, D, B12) and minerals (e.g., iodine and selenium), and other bioactive compounds, including carotenoids with antioxidant properties (e.g., astaxanthin), phytosterols related with hypocholesterolemic effects, and the amino acid taurine, linked to cardioprotective effects [11].

Throughout evolution, the human diet was characterized by a practically even supply of *n*-3 and *n*-6 PUFA, which have played a role in the establishment of genetic patterns (reviewed by Leaf and Weber [12]). However, a significant increase in dietary content of *n*-6 PUFA along with lower *n*-3 PUFA has occurred during the last 100 years, especially in western societies, resulting in *n*-6:*n*-3 PUFA ratios of about 15 to 20, which can promote the pathogenesis of many diseases, such as coronary heart disease (CHD), as suggested by observational and interventional studies [13,14,15]. Indeed, LC-PUFA intake is often dominated by *n*-6 fatty acids, which have been correlated with higher CHD mortality, whereas ideally those should represent only about 20%–40% of total dietary LC-PUFA [14,16,17]. In practice, in 2008, experts from the Food and Agriculture Organization and the World Health Organization (Joint WHO/FAO Expert Consultation on Fats and Fatty Acids in Human Nutrition, Geneva, Switzerland) recommended the daily intake of 250 mg EPA plus DHA in primary prevention, whereas the American Heart Association (AHA) proposed a higher daily dosage (500 mg) for healthy adults [18]. Intake values mentioned for individuals with documented CHD vary between 1 and 2 g per day, whereas up to 4 g EPA and DHA daily have been suggested for hypertriglyceridemic adults [19]. It has been considered by WHO/FAO that convincing evidence exists showing daily consumption of 250 mg (primary prevention) to 2 g (secondary prevention) of EPA and DHA prevents CHD and possibly other degenerative diseases associated with aging, and decreases the risk of fatal CHD events. Although at present the upper level of acceptable micronutrient distribution rate set by WHO/FAO is at 2 g for EPA plus DHA to prevent potential increase in lipid peroxidation, the USA Food and Drug Administration (FDA) has set as safe intake values of 3 g day^−1^, and the former entities have also acknowledged that further research could justify raising the recommended intake value for *n*-3 LC-PUFA in the future. Secondary prevention studies using 850 mg to 4 g EPA and DHA daily have shown significantly reduced total mortality and sudden death (20%–50%) with treatments from 12 to 42 months duration, in patients with CHD or prior myocardial infarction (reviewed by Jacobson [20]). It should be noted that clinical intervention trials involving *n*-3 LC-PUFA intake during limited periods show limited cardiovascular benefits when compared to results from epidemiological studies with populations characterized by a lifetime of consuming diets high in these nutrients [21]. Moreover, variation of the effects of supplementation on primary and secondary prevention of CHD among studies is affected by several factors, including background diet, supplementation means, genetic factors and ethnicity [22].

Several other fields of medicine have explored the potential of these nutrients. They have been implied in the prevention of age-associated decline in cognition, and addressed in the treatment of psychiatric conditions and neurological disorders [23,24]. Cancer research has shown promising results regarding the potential of *n*-3 LC-PUFA in prevention, antitumor effects, adjuvant activity with anticancer drugs, and reduction of cancer therapy-related side effects [25,26]. Another special focus is on fetal neurodevelopment, infant cognitive development and visual acuity, which have been related to maternal seafood consumption and *n*-3 LC-PUFA supply, especially DHA during early life [27,28]. Although it is generally accepted that *n*-3 LC-PUFA play a central role in cell membrane structure and function, cellular signaling and overall physiology, the mechanisms that may lead to a lower occurrence of CVD and other pathologies are yet to be clarified. Fatty acids containing 20 and 22 carbon atoms in their chain, like EPA, arachidonic acid (AA; *n*-6 LC-PUFA), and DHA serve as precursors of eicosanoids (prostaglandins, leukotrienes and thromboxanes). Polyunsaturates and their metabolites are seen with interest by the pharmaceutical industry. They can cause pleiotropic effects in terms of blood rheology, leukocyte function, platelet activation and lipoprotein metabolism, as well as physiological mechanisms like inflammation, vasodilation, blood pressure, pain and fever [29,30]. EPA and DHA are associated with anti-inflammatory effects and may present antiarrhythmic, antithrombotic and antiatherosclerotic properties, improve endothelial function, display mild hypotensive effects, and contribute to reduce blood cholesterol and triacylglycerol (TAG) levels; they may also show hypoglycemic effects [20,21,31]. Since EPA- and AA-derived eicosanoids generally present antagonistic effects, their ratio in tissues, largely dependent on dietary supply, can be often related to cellular signaling, metabolic and health outcomes (reviewed by Simopoulos [13]). Over-reactions mediated by *n*-6 eicosanoids represent main targets for therapeutic drugs, and primary prevention with greater dietary proportions of *n*-3 LC-PUFA may decrease the need for medication [14].

Although more relevance is generally given to supplements delivering *n*-3 LC-PUFA, in order to balance the dietary ratios between *n*-6 and *n*-3 fatty acids, it is generally recommended that formula-fed pre-term infants should include AA as well as DHA, which deposit rapidly in fetal neural tissue during the last months of gestation and the first months of postnatal life. Dietary supply of pre-formed AA and DHA is more likely to meet the requirements of developing tissues, due to limited Δ-6 desaturase activity in humans. In this regard, governmental food safety authorities have determined the safety and approved the use of DHA and AA rich-oils extracted from the single cell organisms *Crypthecodinium cohnii* Javornicky, a marine heterotrophic microalga, and *Mortierella alpina* Peyronel, a soil fungus, respectively, for inclusion as supplements in infant formulas [32]. However, the use of single cell oils may be costly and oils richer in other fatty acids with important physiological roles (e.g., oleic and linoleic acids serving as energy substrates) may still be used in infant formulas. Along with fish oils, microbial oils like those cited have been approved for inclusion in adult foods, including pregnant and nursing women. In particular, vegetarian breast-feeding mothers may benefit from AA supplementation, since this fatty acid is mainly found in animal-based foods (meats, eggs).

Negative effects of elevated LC-PUFA intake may stem from their susceptibility to peroxidation due to high unsaturation degree, and is more likely to occur when antioxidant enzyme activity and micronutrients are lower. Therefore, antioxidant micronutrients (e.g., vitamins E and C, and selenium) are often advised when LC-PUFA supplementation is considered. Whichever their origin, the oxidative instability of LC-PUFA-rich oils requires special attention in order to keep their nutritional quality and avoid the development of off-flavors in final products. When used as food ingredients, the stability of oils may benefit from microencapsulation techniques, the use of antioxidants, and adapted food processing methods [33].

## 3. LC *n*-3 PUFA Sources: The Need for Alternatives

Due to convenience, dietary preferences and other factors, the ability to assess *n*-3 LC-PUFA through supplements or enriched foods is of great interest for most populations with diets low in seafood. According to a review by Ward and Singh [6], a daily dietary intake of 1 g DHA and EPA in seafood would require the consumption of a great amount of fish. Health risks have been associated to the consumption of seafood and fish oils at times, in particular areas, and regarding certain species, and often drive consumers choices away from these products. Indeed, fatty fish and large predatory fish are sometimes the subject of health advisories against the risks of contamination with environmental pollutants (e.g., methyl mercury, dioxins and polychlorinated biphenyls), which are hydrophobic and accumulate along the marine food chain, particularly in lipid depots [34]. Under certain conditions, seafood originating from contaminated regions may be hazardous for humans, particularly pregnant or lactating women and young children, such that advisories to limit consumption of certain types of seafood are released. However, it is generally recognized that, with the exception of a few top predator species, from particularly polluted regions, the benefits provided by seafood to most consumers far outweigh the risks [35,36].

As ingredients for the enrichment of various foods, fish oils show traits that can be found undesirable, the typical odor presenting as a disadvantage for many consumers. Due to odor or off-flavors sometimes derived from lipid peroxidation, these foods may in certain cases lose sensory quality and show a decreased shelf life [37]. At present, they are still the main utilized source of EPA and DHA in *n*-3 LC-PUFA-enriched functional foods. Nonetheless, the market for marine fish oils is largely dominated by the aquaculture industry for use as a main lipid ingredient in feeds for farmed fish. The sustainability of fisheries directed for this purpose has become a major concern as global catches have attained a maximum limit since the 80’s [38]. The availability of this product is affected periodically by environmental conditions, which further contributes to significant rises in the price of this commodity.

The need for sustainable, alternative *n*-3 LC-PUFA sources has stimulated research in several fields. Cellular and molecular methodologies exist that allow the introduction or increased expression of genes for the enzymatic machinery involved in desaturation and elongation of shorter chain fatty acids into LC-PUFA. The potential of plants and animals to biosynthesize these nutrients through genetic manipulation, with the aim to increase the amount of desired fatty acids in their lipid profiles, is currently under investigation with the production of transgenic oilseed crops [39,40] and animals [41,42]. While important advances have been made on the production of oils from transgenic plants, the commercial use of genetically manipulated products in food and feed is the focus of much scientific and social debate and may be delayed due to common negative public perception over this subject.

On the other hand, functional foods enriched with *n*-3 LC-PUFA from either fish or microbial origin, are generally perceived as interesting by consumers [33], as their belief on the effectiveness of these products to deliver important health benefits has increased in recent years [43]. Hence, the *n*-3 LC-PUFA supplemented food sector—including cereals, beverages, cheeses, yogurts, eggs, milk, margarines, spreads and dressings—has been one of the fastest growing food categories in Europe and North America [43]. At present, alternatively to fish oils, some *n*-3 LC-PUFA-rich single cell oils, mostly of microalgal origin, are used in marketed fortified foods. Recently, the health risks and side effects posed by *n*-3 LC-PUFA consumption from various sources were reviewed [37]. Fish oil supplementation was investigated using several delivery methods (capsules, fish-containing meals, fortified foods, or parenteral administration) and results from two studies advised caution when supplying high dietary *n*-3 LC-PUFA levels in both type 2 diabetes mellitus patients and individuals with impaired glucose tolerance, due to risks of increased glycaemia; algal oils appeared to be well tolerated and no major negative effects have been reported, though fishy aftertaste and eructation may occur associated to daily consumption of relatively high (2 g) algal DHA doses [37].

## 4. Microalgae Production

**Figure 1:**
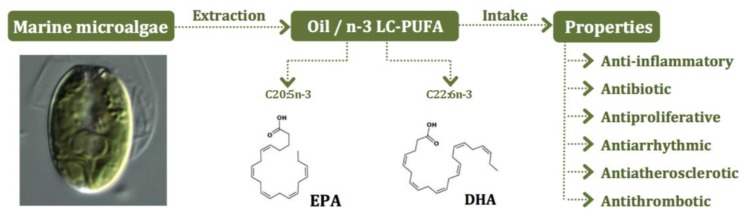
Schematic representation of benefits related with dietary intake of *n*-3 LC-PUFA, obtained by extraction of the oil from marine plankton.

Microalgae offer a promising non-polluted resource for biotechnology and bioengineering of LC-PUFA production (Figure 1), as an alternative to fish oils [5,44]. Compared to terrestrial crop plants, microalgae present a few advantages as *n*-3 LC-PUFA sources, such as commonly occurring genes for the biosynthesis of these nutrients, simpler fatty acid profiles and higher growth rates. In industrial production, outdoor photoautotrophic technologies for microalgae cultures in open pond systems have long been established [45]. Autotrophic microalgal cultivation of marine species in these systems can utilize non-arable lands and water resources considered unsuitable for agriculture; these processes also have a more favorable net energy ratio, relative to heterotrophic systems, but may suffer from unwanted contamination with competitors and predators. Superior growth performance and productivity are naturally obtained using (photo) bioreactors, which present the advantage to provide close control over growth conditions, but imply greater investments related, for example, with energy-dependent expenditures for mixing and cooling. The use of these sophisticated systems is particularly justified regarding high-value products, which are not obtainable in desired quality or quantity from other sources [46]. Heterotrophic cultivation without light, using inexpensive, well-defined mineral medium supplemented with a carbon source is feasible [46]. However, only a few species have been commercially explored and continuous efforts are made in order to diversify the range of microalgal species used [47]. This implies fermentation studies for growth medium optimization, and improvement of culture conditions and systems, in order to maximize the biosynthesis of *n*-3 LC-PUFA in each potential strain [48,49].

Briefly, in order to be selected for commercial oil production with human consumption purposes, microalgae and their products should be non-pathogenic and non-toxic, genetically stable, and present high growth and product formation rates, as reviewed by Ward and Singh [6]. Ideally, for edible oil production, strains used should be capable of accumulating significant amounts of lipids, preferably rich in TAGs high in the LC-PUFA of interest, under heterotrophic or autotrophic growth. The ability to use low-cost fermentation media and growth in low chloride, which has corrosive properties on stainless steel vessels, is also desirable [6].

## 5. Microalgae as Sources of *n*-3 LC-PUFA

In the marine food web, long-chain polyunsaturated fatty acids (≥20 carbon atoms and >3 double bonds) are primarily formed by phytoplankton and transferred onto herbivorous zooplankton, hence affecting food quality for organisms at higher trophic levels [50]. Various auto- and heterotrophic marine species from different classes produce EPA and DHA, whereas AA is generally found in scarce amounts [51,52]. According to recent reviews of total lipid extracts, Bacillariophyceae (diatoms) and Chrysophyceae species may be rich sources of EPA and DHA; Cryptophyceae, Prasinophyceae, Rhodophyceae, Xanthophyceae, Glaucophyceae and Eustigmatophyceae can represent interesting EPA sources, whereas DHA is found in significant amounts mostly in Dinophyceae, Prymnesiophyceae, and Euglenophyceae [53,54]. Interestingly, many species producing LC-PUFA contain low levels of C_18_ fatty acid precursors [5]. The development of commercial processes for microalgal oil production, particularly oils rich in DHA, has benefited from the fact that a number of organisms can accumulate high lipid contents in biomass (up to 50% biomass dry weight; DW), including 30%–70% of this fatty acid [6]. The sector has expanded substantially in recent years and product diversity, in terms of growing processes and subsequent fatty acid profiles, is expected to increase, particularly by developing ingredients with higher EPA and DHA concentration in the near future [55]. Table 1 lists a series of commercially available microalgae oils, including a high-purity, pharmaceutical grade product rich in EPA.

**Table 1:** Levels of eicosapentaenoic acid (EPA) and/or docosahexaenoic acid (DHA) in commercially available oils derived from marine microalgae cultures ^1^.

Company and commercial product designation	% EPA or DHA ^2^	Microbial sources available	Comments
Aurora Algae A2 EPA Pure™	65% EPA (regular) 95% EPA (pharma)	Undisclosed	Phototrophic, open-pond
Qualitas Health EicoOil™	25%–30% EPA	*Nannochloropsis oculata* Hibberd	Phototrophic, open-pond
Algae Biosciences AlgaeBio Omega-3 Origins™	20% EPA; 20% DHA	Undisclosed	Oil blend from two marine strains
DSM-NP life’s DHA™	40%–45% DHA	*Crypthecodinium cohnii* Javornicky	Heterotrophic fermentation
DSM-NP life’s DHA plus EPA™	10% EPA; 22.5% DHA	*Schizochytrium* sp. Goldstein and Belsky	
Lonza DHAid™	35%–40% DHA	*Ulkenia* sp. Gaertner	Heterotrophic fermentation
Source-Omega Source Oil™	35%–40% DHA	*Schizochytrium* sp. Goldstein and Belsky	Heterotrophic fermentation
GCI Nutrients DHA Algae 35% Oil	35% DHA	*Crypthecodinium cohnii* Javornicky	Heterotrophic fermentation

^1^ Information obtained from the respective commercial web sites; ^2^ % EPA or % DHA (m/m) of commercial oil.

**Figure 2:**
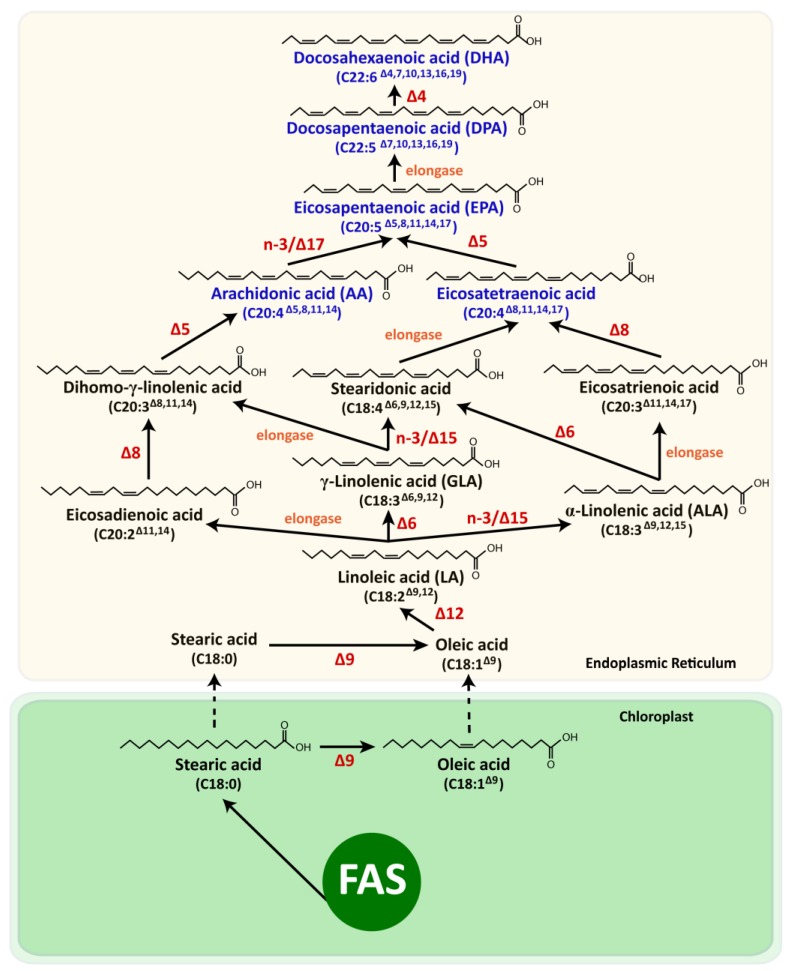
Biosynthetic pathways of the long chain polyunsaturated fatty acids (LC-PUFA) in marine microalgae (except for the polyketide synthase-dependent pathway that is purported to occur in thraustochytrids and coccolithophores). LC-PUFA are painted blue. The desaturases and elongases catalyzing a given step are given in red and orange, respectively. FAS, fatty acid synthase. Unlike mammals, microalgae seldom accumulate high levels of arachidonic acid (AA), as the *n*-3/∆17 desaturase present in the endoplasmic reticulum of these cells converts it to eicosapentaenoic acid (EPA) [5,48,51,60].

LC-PUFA biosynthesis from C_18_ fatty acids, LA and α-linolenic acid (ALA), by the elongase-desaturase pathway is common in microalgae [5]. These usual *n*-6 and *n*-3 pathways involve a desaturation step by Δ6 desaturase, an elongation step and a further desaturation by Δ5 desaturase, yielding AA and EPA (Figure 2). As reviewed by Khozin-Goldberg *et al*. [6], in some species, alternative pathways exist for the biosynthesis of AA and EPA, involving elongation of the C_18_ precursors, followed by Δ8 and Δ5 desaturation. In microalgae, DHA is obtained through EPA elongation into docosapentaenoic acid (DPA) and subsequent desaturation by Δ4 desaturase, or through the anaerobic polyketide synthase (PKS) pathway, as it has been suggested for thraustochytrids [56] and has been inferred *in silico* for the coccolithophore *Emiliania huxleyi* Hay and Mohler [4], which are known for their potential to accumulate important amounts of PUFA [57,58]. These pathways are different from the metabolic synthesis of DHA found in animals, known to occur through the Sprecher’s shunt [59].

Under stressful environmental conditions (e.g., nutrient deficiency, photo-oxidative stress) and in aging algal cells, microalgae tend to stop growth and accumulate neutral lipids, particularly TAGs, which deposit into lipid bodies and are thought to serve as carbon and energy storages for usage under starvation [51,53]. Microalgae species considered oleaginous contain at least 20% lipid of their biomass, and levels can range up to 70% with TAGs accounting for over 90% of the total lipid [53,61]. However, TAGs are usually high in saturated and monounsaturated fatty acids (MUFA) [62], hence, partitioning of LC-PUFA into TAGs is seldom found. EPA, for example, does not generally accumulate significantly in neutral lipid except in some obligate phototrophic microalgae [44]. Usually, LC-PUFA in microalgae are esterified into membrane phospholipids [53], while it is generally thought that esterification into TAGs would provide a more suitable dietary source for the human diet. However, some species may show significant amounts of LC-PUFA esterified into TAG, like DHA in *Thraustochytrium aureum* Goldstein [63], and AA and EPA in the red alga *Porphyridium cruentum* Nägeli [64]. Culture conditions, strains used and growth phase are important determinants of LC-PUFA production and partition into TAGs. Tonon *et al.* [65] described the influence of growth stages on EPA and DHA deposition in TAG extracts in several species. They found a higher percentage of EPA in TAGs of *Nannochloropsis oculata* Hibberd, and of both EPA and DHA in TAGs of *Thalassiosira pseudonana* Hasle and Heimdal and *Pavlova lutheri* Green during the stationary phase. In order to increase microalgae usage for commercial production of LC-PUFA-rich oils, research efforts are directed to the screening and selection of oleaginous microalgal species with the capacity to store LC-PUFA in TAGs, which would allow for more cost-effective production of oil and could also facilitate lipid extraction.

### 5.1. Photoautotrophic Microalgae

It is generally thought that photosynthetic microalgae tend to produce higher levels of EPA than heterotrophs. *Nannochloropsis* Hibberd, *Phaeodactylum* Bohlin, *Nitzschia* Hassall and *Porphyridium* Nägeli can present elevated levels of EPA in total fatty acids, although relatively low cell lipid contents tend to result in small EPA amounts in the biomass (Table 2). Photoautotrophic species contain lipids involved in the photosynthetic metabolism and, unlike various DHA-rich microalgae, the fatty acid profiles of EPA producers usually show other LC-PUFA, like DHA and/or AA [61].

Phototrophic species, such as the eustigmatophyte *Nannochloropsis*, have long been used by the aquaculture industry with the aim to supply larval fish with *n*-3 LC-PUFA through the enrichment of live feeds (e.g., rotifers). In a recent report, Gog *et al*. [66] described a *Nannochloropsis oculata* with a fatty acid profile containing 49% of EPA of total fatty acids, while most commonly *Nannochloropsis* strains show lower levels, in the range of 11%–39% of total fatty acids, varying largely with cultivation conditions (Table 2), particularly nitrogen supply [67,68]. Although *Nannochloropsis* accumulates TAGs in response to environmental stressors, EPA deposition may be low within this lipid class [69], which is instead associated with shorter fatty acids [70]. Deposition of EPA in *Nannochloropsis* seems to be favored by low salinity [68], while total lipids appear to increase in higher salinity waters [69]. The cultivation of *Nannochloropsis* sp. in ultra-dense cultures, with valuable EPA production, using frequent replacement of nutrient medium, has been described [71]. In fact, the genus *Nannochloropsis* is currently the source of marketed oils due to its potential to produce high EPA lipids with very low DHA and AA content, which is advantageous for the manufacture of dietary supplements (Table 1).

**Table 2:** Examples of marine microalgae species characterized by EPA production.

Species	EPA content (% TFA) ^1^	EPA content (% DW) ^2^	References
*Nannochloropsis* sp. Hibberd	38–39	2–3	[72]
	15–18	5–6	[73]
	11–22	3–6	[74]
	15–27	4	[75]
	5–27	2–4	[69]
	30–35	3–4	Soley Biotechnology Institute ^3^
	35–39	4	Soley Biotechnology Institute ^3^
*Phaeodactylum tricornutum* Bohlin	31	5	[76]
	40–57	1–4	[77]
	28	3	[78]
	30–32	3	Soley Biotechnology Institute ^3^
	38–42	4–5	Soley Biotechnology Institute ^3^
*Nitzschia laevis* Hustedt	25–33	3–4	[79]
	11–16	2–3	[80]
*Porphyridium cruentum* Nägeli	25	3	[81]
	41	-	[82]
*Odontella aurita* Agardh	26	-	[83]
*Pavlova lutheri* Green	18–23	-	[83]
	22–29	-	[84]
*Cyclotella cryptica* Lewin and Guillard	17–23	1	[85]
*Cylindrotheca* sp*.* Rabenhorst	24–25	-	[86]

^1^ % TFA, % of total fatty acids; ^2^ % DW, % of biomass dry weight; ^3^ Data obtained from the commercial web site.

The diatom *Phaeodactylum tricornutum* Bohlin is often known for its high EPA content in total fatty acids (Table 2). Differences in preferential deposition of this fatty acid either in TAGs or in polar lipids reflect the importance of cultivation conditions. For example, under optimal conditions tested in a continuous flow reactor, 84% of this fatty acid was found in galactolipids (monogalactosyldiacylglycerols) and 11% EPA in TAGs [87], whereas another experiment varying nitrogen concentration and culture age showed TAGs as the most important EPA reservoir in cells [88]. As EPA is mostly found in the less polar lipid fractions displaying very favorable EPA/AA and EPA/eicosatetraenoic acid ratios, Reis *et al.* [87] suggested that purification of this FA from *P. tricornutum* biomass should be simpler than from other feedstock. Moreover, being amenable to genetic engineering, *P. tricornutum* could be transformed with a glucose transporter gene, allowing the cultivation of this microalga in high cell concentrations under heterotrophic conditions [89].

Another potential EPA producer is *Nitzschia laevis* Hustedt, a diatom that can be grown heterotrophically using glucose and was reported to synthesize more EPA through this process than photosynthetically [90]. Under heterotrophic cultivation, according to Chen *et al.* [91], this microalga can present about 80% neutral lipids of total fatty acids, of which 88% as TAGs. However, only about 6% EPA was determined in the total fatty acids of this lipid fraction. These authors found in a subsequent study that temperature did not affect lipid class proportions, lipid and EPA contents, and reported that TAGs were highly saturated in this species [92].

On the other hand, the red microalga *Porphyridium cruentum* can present 41% EPA of total fatty acids although TAGs appear to represent a minor proportion of all lipid classes (2%) [82]. Some studies have focused on the suitability of this species as a source of highly pure EPA, using various extraction processing and purification protocols [93,94]. Another potential source of EPA-rich oils is the diatom *Odontella aurita* Agardh, which may contain around 26%–28% EPA in its fatty acid profile [94] and is currently approved for use as a food supplement [95], which could facilitate the acceptance of the oil as an ingredient in the market.

Much research on optimization of EPA and lipid production has been conducted at relatively low cell concentrations, in contrast with those needed to support an economical production. As mentioned previously, mass cultivation of phototrophic species in open-pond systems is a relatively low cost system compared to photobioreactors. However, specific growth rates in these systems are relatively low, harvesting may be costly, and the risks of salinity fluctuation and contamination can lead to variable quality and quantity of the final product [96,97]. Cultivation of autotrophic organisms in photobioreactors with high volume to surface ratios is currently limited due to light penetration restrictions, but provides optimized control over environmental parameters, significantly reduces contamination risks, and allows higher biomass concentrations [96]. Overall, application of photosynthetic species for the commercial production of oils requires advances in physiological and genetic engineering studies to enhance growth performances and lipid deposition, optimization of fatty acid composition, biotechnological improvements regarding light capture and contamination, and lowering of costs involved in biomass production and harvesting [5].

### 5.2. Heterotrophic Microalgae

Several marine heterotroph microalgae are considered as good DHA sources (Table 3). These include thraustochytrids from the genera *Thraustochytrium* Sparrow and *Schizochytrium* Goldstein and Belsky, and the dinoflagellate *Crypthecodinium cohnii* Javornicky, which have the advantage to deposit very low or no EPA in cell lipids [61]. These species represent the most preeminent alternative industrial sources of oils rich in DHA (Table 1), with approved use in human foods, especially for application in infant formulas [61,98], since they are considered to be non-pathogenic and nontoxigenic [99].

**Table 3:** Examples of marine microalgae species characterized by DHA production.

Species	DHA content (% TFA) ^1^	DHA content (% DW) ^2^	References
*Schizochytrium mangrovei* Raghuk	31–41	12–21	[100]
*Schizochytrium limacinum* Honda and Yokochi	25–35	5–15	[101]
	-	15–19	[102]
*Schizochytrium* sp. (HX-308) Goldstein and Belsky	40–56	11–20	[103]
*Schizochytrium* sp. Goldstein and Belsky	45–52	20–24	[104]
	28	4	[100]
*Thraustochytrium* sp. Sparrow	23–24	16–17	[105]
*Thraustochytrium aureum* Goldstein	32–37	6–7	[106]
*Thraustochytrium striatum* Schneider	37	2	[100]
*Ulkenia* sp. Gaertner	10–23	5	[107]
*Aurantiochytrium* sp. Yokoyama and Honda	40	18	[108]
*Crypthecodinium cohnii* Javornicky	19–34	2–4	[109]
	63	6	[110]
	53–57	5–6	[111]

^1^ % TFA, % of total fatty acids; ^2^ % DW, % of biomass dry weight.

Ward and Singh [6] reported that certain *Schizochytrium* strains might produce levels as high as 94% DHA of total *n*-3 fatty acids. In addition, *Schizochytrium* sp. has been noted for high resistance to mechanical stirring in fermenter culture [112] and high salinity tolerance [113]. *Schizochytrium* sp. possesses a number of traits that are advantageous for the industry, including high lipid content, elevated DHA production and presentation in the TAG form, as well as good growth in culture under elevated cell concentrations [6]. Nakahara *et al.* [112] reported that in *Schizochytrium* sp. containing about 50% lipids of biomass dry weight, TAGs represented the majority of lipids (93%, m/m) and that 57% of TAGs contained DHA, which accounted for 34% of total fatty acids. Under culture conditions varying carbon, nitrogen and glucose levels in the medium, Yaguchi *et al.* [114] described lipid levels of 78% (mostly neutral, 95%), and 36% DHA of total fatty acids. In *Schizochytrium limacinum* Honda and Yokochi, DHA levels ranged from 34% to 44% of total lipids, along with smaller levels of DPA (5%–7%); higher DHA deposition in TAGs (32%) than in polar lipids was observed at the end of a 10-day culture, though mainly due to drastic decrease in DHA in phospholipids, from 62% to 9% of total fatty acids at day 1 and day 10, respectively [115]. In addition, *Schizochytrium mangrovei* Raghuk harvested during the exponential phase showed elevated amounts of neutral lipids (96%) with 97% TAGs, in which 30% was DHA [116].

An evaluation of various extraction methods using *Thraustochytrium* sp. showed that these organisms are capable to contain over 70% lipids of their dry biomass, including 17% DHA in dry cell weight [105], which makes them ideal for industrial production of DHA-rich oils, as demonstrated in Table 1. *Thraustochytrium aureum* Goldstein was reported to contain TAGs as the main lipid class and to present 40% DHA in total lipids, although cells of this species tended to aggregate in fermenter systems [63]. In a strain of this species, lipid, TAG and PUFA contents appeared to reach the highest values during the early phase of cell growth, with DHA representing about 10% of total lipids [117]. Huang *et al.* [118] described a *Thraustochytrium* sp. strain containing 52% of total fatty acids as DHA, as well as other LC-PUFA (AA, EPA, *n*-6 DPA), which accounted for a total of 76% of total fatty acids.

*Ulkenia* sp. Gaertner, another thraustochytrid, is currently used as a commercial source of DHA-rich TAG oil, which may contain up to 50% DHA and also includes 8%–14% *n*-6 DPA [119]. A native *Thraustochytrid* strain with high similarity to *Ulkenia* sp. showed DHA levels of 10%–24% in total fatty acids [107].

*Ulkenia* sp. and *Schizochytrium* sp. have been considered as much faster growers than *Thraustochytrium* sp. [120], which may be the reason underlying the apparent preferential utilization of the former two in industrial oil production (Table 1). The occurrence of *n*-6 DPA in lipids of these three species [112,113], though in much lower amounts than DHA, was initially seen as a disadvantage for commercial purposes, especially for neonate nutrition applications [59]. The FDA has considered that, since both *n*-3 and *n*-6 DPA are natural components of fish oil, their safety is supported by studies reporting no adverse effects following supplementation with fish or marine oils [119]. It appears that this fatty acid exists naturally in the phospholipids of human cellular membranes [121] and may be retroconverted into AA when dietary supply of the latter is low, as well as contribute for the maintenance of AA when DHA is administered [122]. Thus, inclusion of DPA into the AA + DHA supplements used in infant formulas could actually increase their nutritional value, and these species may provide such suitable oils [61].

The dinoflagellate *Crypthecodinium cohnii* has been used in the commercial production of DHA-rich oils, particularly for inclusion in infant formulas due to its low EPA content, which in high levels can induce bleeding in both infants and nursing mothers [6]. High biomass concentrations in carbon fed batch cultures of *C. cohnii* required prolonged culture times initially, although commercial strains have been selected for rapid growth and acceptable DHA productivities, under high cell concentrations and high shear conditions, with oil production being promoted by nitrogen-deficient conditions (reviewed by Ward and Singh [6]). However, Pleissner *et al.* [109] reported that nutrient limitation (carbon, nitrogen and phosphorus) had no effect on lipid deposition in this species (12%–15%), although wide variations on DHA amounts in total fatty acids were detected (19%–34%). An increase in DHA productivity could result from the use of carbon sources other than glucose, such as acetate or ethanol, though this might imply higher costs and handling challenges (reviewed by Ward and Singh [6]). In a pH-auxostat culture using sodium acetate as the main carbon source, lipid accumulation in excess of 40% was observed, with DHA as half of the total fatty acids found in TAGs [123]. Mendes *et al.* [124] reported DHA levels up to 63% of total fatty acids using carob pulp syrup and yeast extract as carbon and nitrogen sources, respectively, in the culture medium. However, using a supercritical carbon dioxide lipid extraction method, it was demonstrated that DHA contents could be higher than 72% of total fatty acids [125]. Seemingly, few studies have focused on the distribution of DHA among lipid classes in *C. cohnii*. Apparently, this subject is controversial since results vary considerably in the literature [126]. Nonetheless, data related to the industrial production of derived oil mention that 30%–50% DHA is generally presented in TAGs and that these represent about 70% of the extracted oil [32].

Under fermentation, biomass production, lipid composition and DHA yield are generally affected by growth medium composition and other factors, like temperature and salinity [49]. However, the use of fermenters allows for a tight control over these variables, hence allowing the production of high quality commercial oils with better market deliverance guarantees [127]. Other advantages of cultivating organisms in fermenters relate to the ease of product recovery, and protection against weather conditions and diseases, as mentioned previously; but these systems also require the addition of rich media, and productivities are conditioned by the low growth rates of most organisms cultivated [96]. Currently, marketed microalgal DHA-rich oils derive from yet too few species. Nonetheless, heterotrophic microalgae grown in conventional fermenters without the need for light are typically more economic to produce and yield higher biomass than photosynthetic algae cultures [46].

### 5.3. Extraction and Quantification of PUFA in Microalgae

Fatty acid methyl esters (FAME) are commonly assessed by GC-MS (gas chromatography coupled with a mass spectrometry detector) or GC-FID (gas chromatography coupled with a flame ionization detector). However, prior to analysis, lipids must be extracted from microalgal cells, which is a crucial step in the FAME quantification process. For this extraction, there are several well defined procedures including the Bligh & Dyer [128] method and/or Soxhlet extraction. However, these methods have been developed for non-photosynthetic matrices. Microalgae normally possess a thick cell wall, which needs to be disrupted to allow an efficient extraction of lipids otherwise the yield of the lipid extraction may be seriously compromised. This can be achieved by submitting microalgae cells to homogenization with dispersers (e.g., IKA Ultra-Turrax), or other methods that may involve sonication, microwaves, thermolysis, bead-beating, among others [129]. After extraction, the fatty acids must undergo derivatization during which the carboxylic acids are converted into methyl or ethyl esters. This step is needed before GC analysis in order to improve volatility of the fatty acids and achieve better resolutions in the chromatographic procedure [130]. There are several types of derivatization described in the literature, which can be applied to samples of microalgae, such as alkali- and acid-catalyzed transesterification. Previous reports, however, have demonstrated that the use of acid-catalyzed esterification using methanol and acetyl chloride are the most adequate for the typical PUFA usually found in microalgal samples [131]. Upon derivatization, the identification and quantification of PUFA (or any other FAME) is performed in the same way as for lipids from different sources. However, care must be taken that the PUFA profile does not change during the isolation procedure, as it has been shown that in a few diatoms EPA can become depleted if the enzymatic activity is not inhibited by acidification prior to cell disruption [132].

## 6. Future Perspectives and Conclusion

Recent opinion suggests that EPA and DHA should be considered as conditionally essential for humans [133]. The development of an efficient large-scale cultivation system for the commercial production of these nutrients from microalgae would address a major global need [7]. The market for these oils is at the moment limited by supply, hence isolation and exploitation of strains with important *n*-3 LC-PUFA production potential is on-going [47]. Since the microbial route of oil production through fermentation will always be more costly than potential agricultural alternatives (*i.e*., genetically modified crops), continuous efforts must be made in the sense of reducing production costs while focusing on high-value products with applications in human health [61]. Consumers are aware of the importance of an adequate provision of these nutrients and several properties of microalgal oils are particularly appealing, such as their sustainability, high purity and quality, “vegetarian” origin, and improved organoleptic qualities when compared to fish oils. Although the use of genetically modified microalgae by the industry is regarded as a possibility in the process of developing strains producing tailored oils [48], this may, as in other sectors of food production, encounter major opposition from the public opinion. Although genetically modified crops will likely serve as *n*-3 LC-PUFA sources in the future, microalgae oils have a great potential to present purer profiles, which are highly advantageous during processing and may address differentiated purposes in the market.
